# Genetically Informed Regression Analysis: Application to Aggression Prediction by Inattention and Hyperactivity in Children and Adults

**DOI:** 10.1007/s10519-020-10025-9

**Published:** 2020-12-01

**Authors:** Dorret I. Boomsma, Toos C. E. M. van Beijsterveldt, Veronika V. Odintsova, Michael C. Neale, Conor V. Dolan

**Affiliations:** 1 Netherlands Twin Register, Department of Biological Psychology, Vrije Universiteit, Amsterdam, The Netherlands; 2 Amsterdam Public Health (APH) and Amsterdam Reproduction and Development Research Institutes, Amsterdam, The Netherlands; 3 Departments of Psychiatry and Human & Molecular Genetics, Virginia Institute for Psychiatric and Behavioral Genetics, Virginia Commonwealth University, 1-156, P.O. Box 980126, Richmond, VA 23298-0126 USA

**Keywords:** Inattention, Hyperactivity, Aggression, Genetic and environmental prediction, Regression, Structural equation model

## Abstract

We present a procedure to simultaneously fit a genetic covariance structure model and a regression model to multivariate data from mono- and dizygotic twin pairs to test for the prediction of a dependent trait by multiple correlated predictors. We applied the model to aggressive behavior as an outcome trait and investigated the prediction of aggression from inattention (InA) and hyperactivity (HA) in two age groups. Predictions were examined in twins with an average age of 10 years (11,345 pairs), and in adult twins with an average age of 30 years (7433 pairs). All phenotypes were assessed by the same, but age-appropriate, instruments in children and adults. Because of the different genetic architecture of aggression, InA and HA, a model was fitted to these data that specified additive and non-additive genetic factors (A and D) plus common and unique environmental (C and E) influences. Given appropriate identifying constraints, this ADCE model is identified in trivariate data. We obtained different results for the prediction of aggression in children, where HA was the more important predictor, and in adults, where InA was the more important predictor. In children, about 36% of the total aggression variance was explained by the genetic and environmental components of HA and InA. Most of this was explained by the genetic components of HA and InA, i.e., 29.7%, with 22.6% due to the genetic component of HA. In adults, about 21% of the aggression variance was explained. Most was this was again explained by the genetic components of InA and HA (16.2%), with 8.6% due to the genetic component of InA.

**Electronic supplementary material:**

The online version of this article (10.1007/s10519-020-10025-9) contains supplementary material, which is available to authorized users.

## Introduction

Multivariate extensions of the classical twin design, that rest on trait and cross-trait comparisons of resemblances in mono- and dizygotic (MZ and DZ) twins, allow for inferences regarding pleiotropy and correlated environmental effects (Martin and Eaves 1977), the direction of causation between correlated traits (Heath et al. 1993; Duffy and Martin 1994), the moderation of genetic and environmental effects (Purcell 2002), and the analysis of the dimensionality of psychological (psychometric) instruments (Franić et al. 2013). In this contribution, we present an extension of the multivariate twin design that we developed to address questions about prediction of an outcome trait by multiple correlated variables. The model we present involves simultaneously fitting a multivariate genetic covariance structure model to estimate genetic (A, D) and environmental (C, E) variance matrices, and conducting the regression analyses based on the genetic (A, D, or A + D) and environmental (C, E, or C + E) covariances.

We applied the model to measures of aggression, inattention, and hyperactivity that were collected in Dutch twins aged approximately 10 years, and in adult twins. Earlier research has indicated that aggression in children is influenced by genetic and common environmental factors (e.g., Porsch et al. 2016), while measures of attention deficit-hyperactivity disorder (ADHD), inattention, and hyperactivity tend to show strong evidence of non-additive genetic (dominance) influences (Derks et al. 2009). We therefore also investigated the conditions under which a variance decomposition model with both genetic dominance and common environmental influences could be fitted to the data. In univariate applications of the classical twin design, it is hardly ever possible to identify the contributions of both common environmental (C) factors and genetic dominance (D). Ozaki et al. (2011) presented an ACDE model using non-normal Structural Equation Modeling (nnSEM), that includes higher order moments as well as 1st- and 2nd-order moments, in which identification is achieved when not all four (ADCE) latent factors are distributed normally. We focus on identification in multivariate twin data, where identifying constraints can be formulated which allow for estimation of contributions from D and C factors in addition to A and E factors.

We considered the prediction of aggressive behavior by two dimensions of ADHD. ADHD is a neurobiological disorder that is characterized by symptoms of inattention and of hyperactivity/impulsivity, which may manifest in children and in adults. In children, positive associations have been found between broadly defined quantitative measures of aggression and ADHD and attention problems (Biederman et al. 1991; Jensen et al. 1997; Connor et al. 2010; Bartels et al. 2018, see: https://www.action-euproject.eu/ComorbidityChildAggression), and negative associations with academic performance (e.g., Hinshaw, 1992; Hinshaw et al. 2006; Vuoksimaa et al. 2020).

Individual differences in aggression and ADHD are strongly influenced by genetic factors (Derks et al. 2009; Hamshere et al. 2013; Faraone and Larsson, 2019; Odintsova et al. 2019). Studies of the etiology of the association between aggression and ADHD indicated that these associations were largely explained by pleiotropic genetic factors. Hur (2015) presented a review of twin studies on hyperactivity/inattention and Conduct Problems, which showed moderate to high (0.17–0.68) phenotypic correlations, and high genetic correlations (0.43–1.0). Based on a systematic review, Andersson et al. (2020) reported a genetic correlation between externalizing symptoms and ADHD of 0.49 (CI 0.37–0.61). These findings are consistent with the substantial genetic correlation between aggression and ADHD (rg = 1.00, SE = 0.07) that was estimated in a recent meta-analysis of genome-wide association studies of childhood aggression and ADHD (Ip et al. 2019).

Compared to the many studies on aggression and ADHD, a smaller number of analyses have focused on the relationship between aggression and hyperactivity/impulsivity or aggression and inattention. A comprehensive literature review and meta-analysis of studies in children, adolescents and adults on ADHD symptom dimensions indicated that aggressive behavior, and more generally externalizing disorders, are more strongly associated with hyperactivity/impulsivity than with inattention (Willcutt et al. 2012). The developmental trajectories of inattention and hyperactivity are different; young children are more likely to display hyperactive behaviors, while in middle childhood inattentive symptoms become more apparent and tend to persist into adulthood (Franke et al. 2018). Most studies of the association between aggression and ADHD subscales (see Willcutt et al*.* Supplementary Table 9) were done in children. The few publications in adults found no evidence that the associations of externalizing disorders with inattention and with hyperactivity differ.

Inattention and hyperactivity are not independent (e.g., Sokolova et al. 2016). Dolan et al. (2020) employed the classical twin design to analyze the correlation structure among measures of inattention and hyperactivity at the phenotype, genetic and environmental level. Inattention and hyperactivity were assessed by a variety of instruments. They concluded that the strong, broad-sense, genetic effects on inattention and hyperactivity are substantially correlated, regardless of instrument or rater.

Thus, when considering questions such as whether the association with aggression is stronger for inattention than for hyperactivity, we need to take into consideration that these two dimensions are not independent, e.g., there may be genetic pleiotropy, and that associations may differ across age groups. In this contribution, we investigated the differences between inattention and hyperactivity as predictors of aggression in a genetic design, analyzing data from MZ and DZ twins.

## Methods

### Young Participants

The Young Netherlands Twin Register (YNTR) recruits newborn twins and multiples, and follows these children through development by survey studies and dedicated projects in subgroups (Boomsma et al. 2002; van Beijsterveldt et al. 2013; Ligthart et al. 2019). Recruitment of young twins began in 1987 and is ongoing. For the present study, we analyzed data on aggression, hyperactivity and inattention by maternal ratings of twins who were on average 10 years old (mean: 9.94 years; SD: 0.51). The twins were born between 1986 and 2006. In the YNTR, data on aggression were collected from 1995 onwards, and were available for all birth cohorts; data collection for hyperactivity and inattention began later, in 2001, so some twin pairs have incomplete phenotype information. There were 11,345 twin pairs (36% MZ). Table 2 summarizes the total number of participants and the number of missing data by twin member and phenotype.

### Adult Participants

The Adult Netherlands Twin Register (ANTR) began longitudinal data collection by surveys in 1991 from adolescent and adult twins and their relatives. For the current study, we analyzed twin data from ANTR surveys 7 and 8, which were collected between 2004 and 2005 (survey 7), and between 2009 and 2011 (survey 8). The adult twins were on average 29.77 years old (SD: 12.5). The Conners' Adult ADHD Rating Scales (CAARS), which we used to assess inattention and hyperactivity, was first introduced in ANTR survey 7 (Distel et al. 2007). However, this seventh survey did not include an assessment of aggression. ANTR survey 8 (Geels et al. 2013) was collected in two waves. Surveys from the first wave (83% of all responders) included the ASEBA-Adult Self Report aggression scale. The bottom part of Table 3 gives the total number of participants (total number of twin pairs is 7433; 46% MZ) and the number of missing values by twin member and phenotype. In contrast to the child data, the adult dataset had a substantial number of incomplete twin pairs (32% for MZ pairs and 51% for DZ pairs).

### Zygosity Assessment

Most YNTR and ANTR surveys include a set of items concerning the twins' physical resemblance and the degree to which the twins, in childhood, were confused by parents, other relatives, and strangers. In the YNTR and ANTR data, discriminant analyses were performed to assess the accuracy of zygosity classification based on survey items, using information from blood group and DNA polymorphisms as the index of true zygosity (Ligthart et al. 2019). In both the YNTR and ANTR, the accuracy of classification was high, ranging between 92 and 96%, depending on age and rater. In 31% of same-sex young twins and in the majority of same-sex ANTR twins (59%) zygosity assessment was based on DNA information.

### YNTR Phenotyping

The Child Behavior Checklist (CBCL) is a standardized questionnaire designed for parents to report the frequency and intensity of their children’s behavioral and emotional problems (Achenbach et al. 2017). It is part of the Achenbach System of Empirically Based Assessment (ASEBA: https://aseba.org/), and consists of 120 items, which are rated on a 3-point scale. The response options range from ‘‘not true = 0’’, ‘‘somewhat or sometimes true = 1’’, to ‘‘very true or often true = 2’’. The Aggression Problem subscale contains 18 items; the total aggression score ranges from 0 to 36, allowing for up to 3 missing items (van Beijsterveldt et al. 2003). The Conners’ Parent Rating Scale-Revised (CPRS-R; Conners 2001; Conners et al. 1998; Derks et al. 2008) also assesses behavioral problems in children by parental ratings. The short version contains 27 items, which are rated on a 4-point scale, ranging from ‘‘not true at all = 0’’ to ‘‘very much true = 3’’. The two CPRS-R subscales that measure hyperactivity and inattention consist of 6 items each, allowing for 1 missing item per subscale. The phenotypic scores range from 0 to 18. The CPRS-R has good internal and test–retest reliability (Faries et al. 2001).

### ANTR Phenotyping

The adult twins completed the ASEBA Adult Self Report (ASR) (Achenbach et al. 2017), which includes 15 aggression items that are rated on a 3-point scale, allowing for 3 missing items. The resulting aggression scores range from 0 to 30 (Hagenbeek et al. 2018). The Conners' Adult ADHD Rating Scales screening self-report (CAARS—S:SV) includes two 9-item subscales for the quantitative assessment of inattentive symptoms (inattention) and hyperactive-impulsive symptoms (hyperactivity). There are no items common to the subscales. The items in the inattention and hyperactivity scales correspond to the symptoms that represent the diagnostic criteria of adult ADHD as outlined in DSM-IV-TR. All items were scored on a scale from ‘‘not true at all = 0’’ to ‘‘very much true = 3’’. The sum score of each subscale ranged from 0 to 27. Missing items were handled as per CAARS instructions (Conners et al. 1999; Saviouk et al. 2011) which allows the scoring of scales with up to two missing items.

### Statistical Analyses

Statistical analyses were carried out in OpenMx (Neale et al. 2016) using full information maximum likelihood (ML) estimation. In all models, sex and age were included as fixed effects (the OpenMx scripts are given in the Online Appendix).

#### Phenotypic Regression of Aggression on Hyperactivity and Inattention

**Fig. 1:**
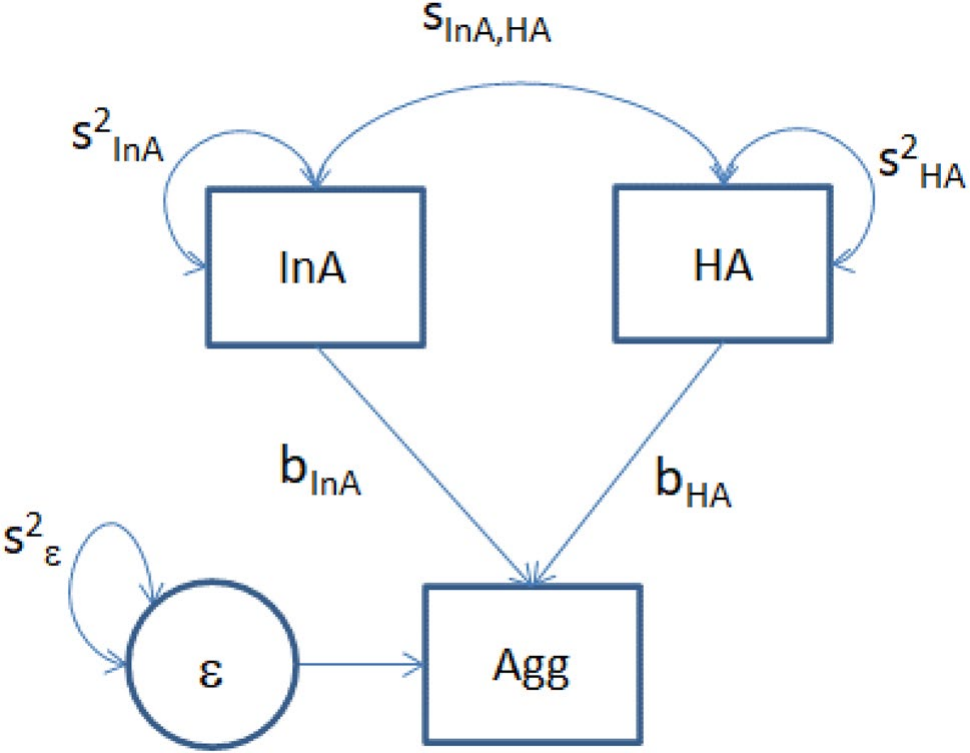
Regression of aggression (Agg) on the correlated variables inattention (InA) and hyperactivity (HA). The covariates sex and age are not depicted

We first carried out the phenotypic analyses, in which we regressed aggression (Agg) on sex, age, hyperactivity (HA) and inattention (InA), in the child and in the adult cohort. The within-person regression model, depicted in Fig. 1 (where the fixed effects of sex and age are left out), is:

$$  {\text{Agg}}_{{\text{i}}}  = {\text{b}}_{0}  + {\text{b}}_{{{\text{Sex}}}} *{\text{Sex}}_{{\text{i}}}  + {\text{b}}_{{{\text{Age}}}} *{\text{Age}}_{{\text{i}}}  + {\text{b}}_{{{\text{HA}}}} *{\text{HA}}_{{\text{i}}}  + {\text{b}}_{{{\text{InA}}}} *{\text{InA}}_{{\text{i}}}  + \varepsilon _{i} , $$

where the subscript i denotes person, and ε is the prediction error (regression residual). Conditional on sex and age, the phenotypic aggression variance (${\text{s}}^{{2}}_{{{\text{Agg}}|{\text{age}},{\text{sex}}}}$) was decomposed into four parts:

$$ {\text{s}}^{{2}}_{{{\text{Agg}}|{\text{age}},{\text{sex}}}} = {\text{b}}_{{{\text{HA}}}}^{{2}} *{\text{s}}^{{2}}_{{{\text{HA}}}} + {\text{b}}_{{{\text{InA}}}}^{{2}} *{\text{s}}^{{2}}_{{{\text{InA}}}} + {2}*{\text{b}}_{{{\text{HA}}}} *{\text{b}}_{{{\text{InA}}}} *{\text{s}}_{{{\text{HA}},{\text{InA}}}} + {\text{s}}^{{2}}_{\varepsilon } $$

The variance terms ${\text{b}}_{{{\text{HA}}}}^{{2}} *{\text{s}}^{{2}}_{{{\text{HA}}}} {\text{and b}}_{{{\text{InA}}}}^{{2}} *{\text{s}}^{{2}}_{{{\text{InA}}}}$ can be attributed to hyperactivity and inattention, respectively. However, the term ${2}*{\text{b}}_{{{\text{HA}}}} *{\text{b}}_{{{\text{InA}}}} *{\text{s}}_{{{\text{HA}},{\text{InA}}}}$ which arises when HA and InA are correlated, cannot be attributed unambiguously to either. We therefore report these three variance components separately. We fitted the phenotypic regression models in OpenMx (Neale et al. 2016) to the all twin data, regardless of the patterns of missingness. In these analyses we constrained the regression to be equal over MZ and DZ groups and over twin 1 and twin 2 (the two twins in a pair) and left the MZ twin 1–MZ twin 2 covariances and the DZ twin 1–DZ twin 2 covariances unconstrained, to accommodate the dependence of the MZ and DZ twin data (Neale et al. 1994). Below, we report the standardized regression coefficients, and the decomposition of the standardized variance of aggression.

#### Genetic Modeling: ADCE Twin Model

We first calculated the MZ and DZ 6 × 6 phenotypic covariance matrices, whose standardized solution is given to describe MZ and DZ twin resemblances and fitted a trivariate Cholesky decomposition to the data (Brezinski 2005) to estimate genetic and environmental covariance matrices for aggression, hyperactivity and inattention. The phenotypic (3 × 3) covariance matrix of the phenotypes, conditional on age and sex ($\sum_{{{\mathbf{ph}}|{\mathbf{age}},{\mathbf{sex}}}}$) was decomposed into the following four covariance matrices (Martin and Eaves 1977; Franić et al. 2012):

$$ \begin{gathered} {\text{In the 1}}0{\text{ year olds}}:{\sum}_{{{\mathbf{ph}}|{\mathbf{age}},{\mathbf{sex}}}} = {\sum}_{{\mathbf{A}}} + {\sum}_{{\mathbf{C}}} + {\sum}_{{\mathbf{D}}} + {\sum}_{{\mathbf{E}}} , \hfill \\ {\text{and in the adults}}:{\sum}_{{{\mathbf{ph}}|{\mathbf{age}},{\mathbf{sex}}}} = {\sum}_{{\mathbf{A}}} + {\sum}_{{\mathbf{D}}} + {\sum}_{{\mathbf{E}}} , \hfill \\ \end{gathered} $$

where Σ_**A**_ is the additive genetic covariance matrix, Σ_**D**_ the dominance genetic covariance matrix, Σ_**C**_ the common (shared between twins) environmental covariance matrix, and Σ_**E**_ the unique (unshared) environmental covariance matrix. To render the model identified in the 10-year olds, we added identifying constraints (informed by the MZ and DZ twin phenotypic correlations; see Tables 2 and 3). The 3 × 3 covariance matrix Σ_**C**_, which was included to accommodate the contribution of shared environmental influences to aggression, was specified as follows:

$$ {\varvec{\Sigma}}_{\mathbf{C}}^{\,} = {\varvec{\Lambda}}_{\mathbf{C}}^{\,} {\varvec{\Lambda}}_{\mathbf{C}}^{\mathbf{t}},$$

where **t** denotes transpose and **Λ**_**C**_

$$ {{\varvec{\Lambda}}}_{C} = \begin{array}{*{20}c} {} & {Agg} & {HA} & {InA} \\ {Agg} & {c_{11} } & 0 & 0 \\ {HA} & {c_{21} } & 0 & 0 \\ {InA} & {c_{31} } & 0 & 0 \\ \end{array} $$

The parameter c_11_ expresses the common environmental influences on aggression. We included the parameters c_21_ and c_31_ to accommodate shared environmental effects, if any, that are common to all three phenotypes. The 3 × 3 covariance matrix Σ_**D**_ was modeled as **Λ**_**D**_**Λ**_**D**_^t^, where

$$ {{\varvec{\Lambda}}}_{{\mathbf{D}}} = \begin{array}{*{20}c} {} & {Agg} & {HA} & {InA} \\ {Agg} & 0 & 0 & 0 \\ {HA} & 0 & {d_{22} } & 0 \\ {InA} & 0 & {d_{32} } & {d_{33} } \\ \end{array} $$

This is the Cholesky decomposition, with the dominance effects limited to hyperactivity and inattention. In both groups (children and adults), we modeled the additive genetic 3 × 3 covariance matrix Σ_**A**_ and the unshared environmental covariance matrix Σ_**E**_ as **Λ**_**A**_**Λ**_**A**_^t^ and **Λ**_**E**_**Λ**_**E**_^t^, respectively, where

$$ {{\varvec{\Lambda}}}_{{\mathbf{A}}} = \begin{array}{*{20}c} {} & {Agg} & {HA} & {InA} \\ {Agg} & {a_{11} } & 0 & 0 \\ {HA} & {a_{21} } & {a_{22} } & 0 \\ {InA} & {a_{31} } & {a_{32} } & {a_{33} } \\ \end{array} $$

$$ {{\varvec{\Lambda}}}_{{\mathbf{E}}} = \begin{array}{*{20}c} {} & {Agg} & {HA} & {InA} \\ {Agg} & {e_{11} } & 0 & 0 \\ {HA} & {e_{21} } & {e_{22} } & 0 \\ {InA} & {e_{31} } & {e_{32} } & {e_{33} } \\ \end{array} $$

That is, **Λ**_**A**_ and **Λ**_**E**_ were obtained from the full 3 × 3 Cholesky decomposition. The parameters were estimated by modeling the MZ and DZ twin covariance matrices (6 × 6: 3 traits in two twins), conditional on age and sex:

$$ \begin{array}{*{20}l} {} & {\text{MZ twin 1}} & {\text{MZ twin 2}} \\ {\text{MZ twin 1}} & {\Sigma_{\text{A}} + \Sigma_{\text{C}} + \Sigma_{\text{D}} + \Sigma_{\text{E}} } & {\Sigma_{\text{A}} + \Sigma_{\text{C}} + \Sigma_{\text{D}} } \\ {\text{MZ twin 2}} & {\Sigma_{\text{A}} + \Sigma_{\text{C}} + \Sigma_{\text{D}} } & {\Sigma_{\text{A}} + \Sigma_{\text{C}} + \Sigma_{\text{D}} + \Sigma_{\text{E}} } \\ \end{array} $$

$$ \begin{array}{*{20}l} {} & {\text{DZ twin 1}} & {\text{DZ twin 2}} \\ {\text{DZ twin 1}} & {{{\Sigma}}_{{\text{A}}}+{\Sigma }_{{\text{C}}} +{\Sigma }_{{\text{D}}}+ {\Sigma }_{{\text{E}}}} & {{.5*\Sigma }_{{\text{A}}} + {\Sigma }_{{\text{C}}}  + {.25*\Sigma }_{{\text{D}}} } \\ {\text{DZ twin 2}} & {{.5*\Sigma}_{{\text{A}}} + {\Sigma}_{{\text{C}}} + {.25*\Sigma}_{{\text{D}}}} & {{{\Sigma}}_{{\text{A}}} + {\Sigma}_{{\text{C}}} + {\Sigma}_{{\text{D}}} + {\Sigma}_{{\text{E}}} } \\ \end{array} $$

where $\Sigma_{A} ,\Sigma_{C} ,\Sigma_{D} \,{\text{and}}\;\Sigma_{E}$, are defined as above. We calculated the total genetic covariance matrix $\Sigma_{G} = \Sigma_{A} + \Sigma_{D}$ and the total environmental covariance matrix $\Sigma_{T} = \Sigma_{C} + \Sigma_{E}$ (in the adults, this is $\Sigma_{T} = \Sigma_{E}$).

#### Genetic Modeling: A + D, C + E Regression Models

The trivariate genetic modeling provided an insight into the genetic and non-genetic correlations of hyperactivity and inattention with aggression, but did not address explicitly the question which of the subscales HA and InA is the stronger predictor of aggression. We included the regression of aggression on hyperactivity and inattention at the level of the genetic Σ_G_ and the environmental covariance matrix Σ_T_, where Σ_G_ equals $\Sigma_{A} + \Sigma_{D} ,$ and Σ_T_ equals $\Sigma_{C} + \Sigma_{E}$. In the adults, we have $\Sigma_{T} = \Sigma_{E}$. We did not attempt to conduct the regression analysis at the level of the individual (A,D, C and E) covariance matrices because Σ_C_ and Σ_D_ are positive semi-definite by definition (i.e., rank 1 and rank 2, respectively). In addition, Σ_A_ was found to be positive semi-definite (rank 1) in the children. We therefore defined covariance matrices Σ_G_ and Σ_T_ to conduct the regression analyses at the total genetic (G) and the total (T) environmental level. Specifically, given Σ_G_ (i.e., $\Sigma_{A} + \Sigma_{D}$),
we partitioned the matrix into the following matrices:
i.e., the genetic covariance matrix of hyperactivity and inattention, and
i.e., the genetic covariance of aggression with hyperactivity and inattention. We calculated the genetic regression coefficients ${\mathbf{b}}_{{\text{G}}} = \left[ {{\text{b}}_{{{\text{G}}\_{\text{HA}}}} ,{\text{ b}}_{{{\text{G}}\_{\text{InA}}}} } \right]^{{\text{t}}}$ as follows: ${\mathbf{b}}_{{\text{G}}} = \Sigma_{{{\text{G2}}}}^{{ - {1}}} \Sigma_{{{\text{G1}}}} .$ The decomposition of genetic variance associated with the genetic regression model is:
where s_G_^2^__ε_ is the genetic prediction error variance. Using the same approach, we calculated ${\mathbf{b}}_{{\text{T}}} = \, \left[ {{\text{b}}_{{{\text{T}}\_{\text{HA}}}} ,{\text{ b}}_{{{\text{T}}\_{\text{InA}}}} } \right]^{{\text{t}}}$ as follows ${\mathbf{b}}_{{\text{T}}} = \Sigma_{{{\text{T2}}}}^{{ - {1}}} \Sigma_{{{\text{T1}}}} ,$ and obtained the decomposition of total environmental variance: 
Given estimates of the phenotypic, genetic, and environmental variance components, we standardized these by dividing by the total phenotypic, genetic, and environmental variance. The results of main interest are the genetic and environmental variance components standardized by the total phenotypic variance, as these reveal the relative contributions of genetic and environmental factors to the phenotypic regression of aggression on hyperactivity and inattention. Table 1 contains a summary of the decompositions of variance in the regression models.

**Table 1:**
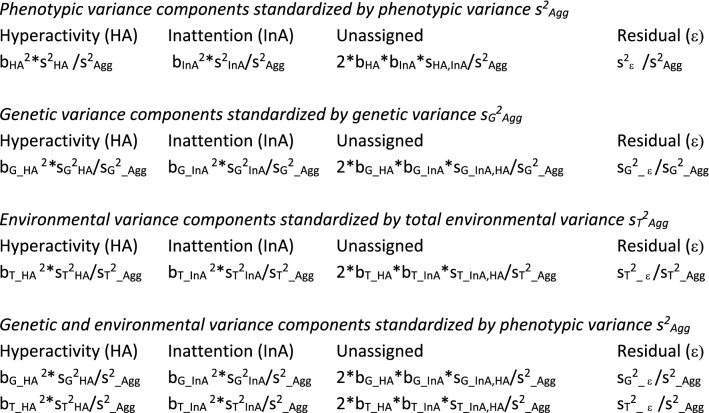
Variance components based on regression analysis with aggression as outcome and hyperactivity and inattention as predictors: variance components due to hyperactivity, inattention, the covariance of hyperactivity and inattention (unassigned to either hyperactivity or inattention) and a residual term

*b* = regression coefficient, *s*^2^ = total phenotypic variance, *s*_G_^2^ = (broad-sense) genetic variance, *s*_*T*_^2^ = total environmental variance

## Results

### Descriptive Statistics and Phenotypic Regression Analysis

Tables 2 and 3 contain the MZ and DZ correlation matrices, standard deviations, sample sizes and the number of missing values in the child and in the adult sample. In the children (Table 2), the phenotypic correlations among the three traits revealed that the correlation of Agg with HA (~ 0.60) is higher than the correlation of Agg with InA (~ 0.45), while the correlation between the two predictors InA and HA is ~ 0.61. In the adults (Table 3), we note that the correlations are appreciable lower. The correlations of Agg with HA are between ~ 0.29 and ~ 0.36 and the correlations of Agg with InA are consistently greater, between ~ 0.35 and ~ 0.48. The correlation between the predictors InA and HA varies between ~ 0.40 and ~ 0.46. Based on these results, it would seem that in the children HA may be the stronger predictor of Agg, while in the adults, InA is the stronger predictor. However, it is important to note that in adults hyperactivity and inattention were assessed four years before aggression was measured, which may have influenced the results.

**Table 2:** Children (10-year olds)

	Agg1	InA1	HA1	Agg2	InA2	HA2
MZ						
Agg1	1.000					
InA1	0.456	1.000				
HA1	0.605	0.611	1.000			
Agg2	**0.797**	0.381	0.504	1.000		
InA2	0.385	**0.722**	0.483	0.443	1.000	
HA2	0.518	0.497	**0.772**	0.587	0.620	1.000
SD	4.772	3.798	2.905	4.601	3.829	2.934
DZ						
Agg1	1.000					
InA1	0.454	1.000				
HA1	0.587	0.615	1.000			
Agg2	**0.442**	0.230	0.271	1.000		
InA2	0.232	**0.186**	0.227	0.439	1.000	
HA2	0.254	0.208	**0.276**	0.582	0.620	1.000
SD	4.865	4.065	3.081	4.682	4.072	3.204

		Missing values (individuals) for twin1 and twin2					
		Agg1	InA1	HA1	Agg2	InA2	HA2
Total sample size (number of pairs) and marginal number of missing* values							
MZ	4209	33	1125	1115	65	1120	1113
DZ	7136	33	1703	1696	100	1727	1712

MZ and DZ correlation matrices of within-person, twin correlations (in bold) and cross-twin cross-trait correlations and standard deviations (corrected for sex and age) for Aggression (Agg), Inattention (InA) and hyperactivity (HA) in twin 1 and twin 2

*SD* standard deviation

*In the earlier birth cohorts data on InA and HA were not collected

**Table 3:** Adults

	Agg1	InA1	HA1	Agg2	InA2	HA2
MZ						
Agg1	1.000					
InA1	0.348	1.000				
HA1	0.310	0.421	1.000			
Agg2	**0.460**	0.224	0.199	1.000		
InA2	0.227	**0.425**	0.160	0.391	1.000	
HA2	0.173	0.220	**0.365**	0.290	0.460	1.000
SD	3.338	3.568	3.474	3.265	3.683	3.405
DZ						
Agg1	1.000					
InA1	0.479	1.000				
HA1	0.360	0.440	1.000			
Agg2	**0.172**	0.189	0.010	1.000		
InA2	0.053	**0.203**	0.089	0.406	1.000	
HA2	-0.010	0.138	**0.128**	0.319	0.395	1.000
SD	3.373	3.861	3.559	3.443	3.746	3.317

		Missing values (individuals) for twin1 and twin2					
		Agg1	InA1	HA1	Agg2	InA2	HA2
Total sample size (number of pairs) and marginal number of missing* values							
MZ	3438	1689	1351	1350	1689	1462	1689
DZ	3995	2284	1976	1973	2332	2166	2332

MZ and DZ correlation matrices of within-person, twin correlations (in bold) and cross-twin cross-trait correlations and standard deviations (corrected for sex and age) for Aggression (Agg), Inattention (InA) and hyperactivity (HA) in twin 1 and twin 2

*SD* standard deviation

*Data collection for InA and HA preceded data collection for Aggression by 4 years; for InA and HA data collection took place in ANTR survey 7, for Aggression in ANTR survey 8

We conducted phenotypic regression analyses on the basis of the within person phenotypic covariance (Agg-InA-HA) matrices. Results for these phenotypic analyses are summarized in Table 4A, which includes the standardized variance decomposition conditional on age and sex. Based on these results, Table 4B presents the proportions of explained phenotypic variance in aggression by the main effects of InA and HA, and by their covariance.

**Table 4:** Phenotypic regression results in children and adults, with Aggression (Agg) as outcome and Inattention (InA) and Hyperactivity (HA) as predictors, where:${\text{Agg}}_{{\text{i}}} {\text{ }} = {\text{ b}}_{0}  + {\text{b}}_{{{\text{Sex}}}} {\text{*Sex}}_{{\text{i}}}  + {\text{b}}_{{{\text{Age}}}} {\text{*Age}}_{{\text{i}}}  + {\text{b}}_{{{\text{InA}}}} {\text{*InA}}_{{\text{i}}}  + {\text{b}}_{{{\text{HA}}}} {\text{*HA}}_{{\text{i}}} {\text{ + }}\varepsilon _{{\text{i}}} ,$

A. Raw regression coefficients, with standard errors (se) or CI-95 intervals in parentheses			
	Agg	InA	HA
Children			
Intercept **(b**_**0**_**)**	0.044 (se 0.890)	5.23 (se 0.700)	5.38 (se 0.567)
Sex **(b**_**Sex**_**)**	− 1.18 (se 0.063)	− 1.47 (se 0.063)	− 1.33 (se 0.047)
Age **(b**_**Age**_**)**	0.050 (se 0.089)	− 0.086 (se 0.070)	− 0.235 (se 0.057)
Phenotypic Agg on InA **(b**_**InA**_**)**	0.165 (0.154–0.184)		
Phenotypic Agg on HA **(b**_**HA**_**)**	0.773 (0.758–0.798)		
Adults			
Intercept **(b**_**0**_**)**	3.86 (se 0.126)	7.57 (se.134)	7.58 (se 0.123)
Sex **(b**_**Sex**_**)**	0.727 (se 0.089)	− 0.244 (se.093)	0.050 (se 0.086)
Age **(b**_**Age**_**)**	− 0.036 (se 0.003)	− 0.035 (se .003)	− 0.010 (se 0.003)
Phenotypic Agg on InA **(b**_**InA**_**)**	0.293 (0.273–0.300)		
Phenotypic Agg on HA **(b**_**HA**_**)**	0.173 (0.138–0.174)		

B. Proportions of explained phenotypic variance of Agg with CI-95 intervals in parentheses			
	b_InA_^2^*s^2^_InA_/s^2^_Agg_	b_HA_^2^*s^2^_HA_ /s^2^_Agg_	2*b_HA_*b_InA_*s_HA,InA_/s^2^_Agg_
Kids: Proportion of variance	0.019 (0.016–0.024)	0.252 (0.236–0.267)	0.085 (0.078–0.094)
Adults: Proportion of variance	0.106 (0.093–0.107)	0.031 (0.019–0.031)	0.049 (0.044–0.051)

s^2^ stands for variance and s represents standard deviation; s_HA,InA_ is the covariance of InA and HA

We first discuss the results in the children, where we note a consistent effect of sex. On average, girls scored lower than boys on all three phenotypes. The effects of age on Agg and InA were not significant (judging by the standard errors), but there was an effect of age on HA (α = 0.05). Even in this sample with limited variation in age (average age: 9.94 years; SD: 0.51), HA decreased with age, indicating fewer HA problems as children grow up (b_Age_ =  − 0.235, se = 0.057). Overall, sex and age combined explained about 1.9%, 3.6%, and 4.4% of the phenotypic variance of Agg, InA, and HA, respectively. Conditional on sex and age, we obtained regression coefficients of 0.165 (CI-95: 0.154–0.184 for InA) and 0.773 (CI-95: 0.758–0.798 for HA) in the regression of Agg on InA and HA. The total explained variance was 35.6%: InA explained 1.9% (CI-95: 1.6–2.4%) and HA explained 25.2% (CI-95: 23.6–26.7%) of the phenotypic Agg variance. The component due to the covariance between InA and HA explained an additional 8.5% (CI-95: 7.8–9.4%). Clearly HA emerged as the better phenotypic predictor, accounting for 25.2%/35.6% =  ~ 71% of the explained variance, with InA accounting for 1.9%/35.6% =  ~ 5% and the covariance of InA and HA accounting for 8.5%/35.6% =  ~ 24% of the explained variance.

In the adults, we note a significant effect of sex on Agg and InA (α = 0.05), but no sex effect on HA. On average females scored higher on Agg and lower on InA. The effect of age was consistently negative, indicating lower scores with increasing age. Overall, sex and age combined explained 2.8%, 1.4%, and 0.1% of the phenotypic variance of Agg, InA, and HA, respectively. Conditional on sex and age, we obtained regression coefficients of 0.293 (CI-95: 0.273–0.300; InA) and 0.173 (CI-95: 0.138–0.174; HA) in the regression of Agg on InA and HA. The total explained variance was 18.6%: InA explained 10.6% (CI-95: 9.3–10.7%) and HA explained 3.1% (CI-95: 1.9–3.1%) of the phenotypic Agg variance. The component due to the covariance between InA and HA explained an additional 4.9% (CI-95: 4.4–5.1%). These results suggest that InA is the better phenotypic predictor in the adults, accounting to 10.6%/18.6% = 57% of the explained variance with HA and the covariance of HA accounting for 3.1%/18.6% = 17% and 4.9%/18.6% = 26%, respectively. However, the 4-year interval between the assessment of Agg and ADHD should be considered when interpreting these results.

### Combined Genetic Covariance Structure and Regression Analyses in 10-Year Olds

Table 2 presents the correlations between twins and among scales in children. The MZ twin correlations were 0.79 (Agg), 0.72 (InA), and 0.77 (HA). The DZ correlations were substantially lower: 0.44 (Agg), 0.18 (InA), and 0.27 (HA). In the genetic covariance structure model, we therefore included additive genetic (A), dominance genetic (D), common environmental (C) and unique environmental (E) components (see model specification of matrices above). Based on twin data from mono- and dizygotic twins the full ADCE model is not identified and constraints as outlined above were applied to the 3 × 3 C and D matrices. The estimates obtained in fitting the ADCE model are given in Table 5.

**Table 5:** Estimates of additive and dominance genetic and common and unique environmental variance–covariance matrices (Σ), based on fitting an ADCE model to the trivariate twin data, conditional on age and sex

Child	ΣA			ΣD			ΣC			ΣE		
	Agg	InA	HA	Agg	InA	HA	Agg	InA	HA	Agg	InA	HA
Agg	16.20	1	1	–	–	–	1.86	1	1	4.50	0.258	0.392
InA	6.66	2.77	1	–	8.84	0.526	0.66	0.23	1	1.28	4.21	0.422
HA	7.00	2.89	3.02	–	3.28	4.40	0.44	0.16	0.10	1.17	1.22	1.98

	h^2^			d^2^			c^2^			e^2^		
	0.717	0.172	0.382	–	0.550	0.462	0.082	0.014	0.011	0.199	0.262	0.208

Adult	ΣA			ΣD			ΣE		
	Agg	InA	HA	Agg	InA	HA	Agg	InA	HA
Agg	2.36	0.584	0.814	2.94	0.546	0.814	5.95	0.281	0.224
InA	1.88	4.36	0.831	1.24	1.76	− 0.041	1.91	7.69	0.402
HA	0.08	2.52	2.52	2.10	− 0.08	2.26	1.50	3.05	7.49

	h^2^			d^2^			e^2^		
	0.209	0.315	0.117	0.260	0.127	0.190	0.528	0.556	0.631

The lower triangles give (co)variances, and the upper triangle correlations. The last row (for children and for adults) contains the standardized variance components (h^2^ is heritability; d^2^, c^2^ and e^2^ give the proportions of variance explained by D, C and E)

Heritability of Agg was 71% and common environment shared by twins accounted for 8% of the phenotype Agg variance. The estimates for the total heritability of HA and InA were also high, with relatively large contributions of genetic dominance. The broad-sense heritability of HA was 17% (A) + 55% (D) = 72% and of InA 38% + 46% = 74%. Common environmental influences (shared with aggression) accounted for 1.4% and 1.1% of the variance of HA and InA, respectively. The C variance–covariance matrix is rank 1, which follows from our model specifications by definition, and matrix D is rank 2, again by definition. We found that matrix A is almost rank 1 (eigenvalues = 21.99, 0.006, ~ 0.00) and matrix E is rank 3. As mentioned above, given the ranks of these matrices, it is not possible to assess the regression model at the level of A, D and C (the covariance matrices of the predictors are singular). We therefore based the regression analyses on total genetic effects (broad sense: G = A + D) and the total environmental effects (T = C + E). The A + D and C + E matrices both have rank 3. The estimates of the regression coefficients, for the phenotypic, genetic and non-genetic components in our model, are given in Table 6.

**Table 6:** Genetic (G(A + D)) and environmental (T(C + E)) regression results in children, where for both the genetic and environmental regression ${\text{Agg}}_{{\text{i}}} = {\text{b}}_{0} + {\text{b}}_{{{\text{Sex}}}} *{\text{Sex}}_{{\text{i}}} + {\text{b}}_{{{\text{Age}}}} *{\text{Age}}_{{\text{i}}} + {\text{b}}_{{{\text{InA}}}} *{\text{InA}}_{{\text{i}}} + {\text{b}}_{{{\text{HA}}}} *{\text{HA}}_{{\text{i}}} + \varepsilon_{{\text{i}}}$

Child	Agg	InA	HA
Raw regression coefficients with standard errors (se) or CI-95 intervals in parentheses			
Intercept (**b**_**0**_**)**	0.047 (se 0.760)	5.28 (se 0.705)	5.39 (se 0.515)
Sex (**b**_**sex**_**)**	− 1.18 (se 0.063)	− 1.49 (se0.063)	− 1.33 (se 0.047)
Age (**b**_**Age**_**)**	0.050 (se 0.076)	− 0.090 (se 0.071)	− 0.237 (se 0.052)
G(A + D) Agg on InA (**b**_**InA**_**)**	0.134 (0.091–0.174)		
G(A + D) Agg on HA (**b**_**HA**_**)**	0.830 (0.778–0.881)		
T(C + E) Agg on InA (**b**_**InA**_**)**	0.205 (0.149–0.268)		
T(C + E) Agg on HA (**b**_**HA**_**)**	0.636 (0.532–0.744)		

Proportions of explained phenotypic variance of Agg with CI-95 intervals in parentheses				
	Total genetic	b_G_InA_^2^*s_G_^2^_InA_/s_G_^2^__Agg_	b_G_HA_^2^ *s_G_^2^_HA_/s_G_^2^__Agg_	2*b_G_HA_*b_G_InA_*s_G_InA,HA_/s_G_^2^__Agg_
Explained by G(A + D)	0.414	0.013 (0.006–0.018)	0.316 (0.275–0.358)	0.085 (0.060–0.108)
	Total environ.	b_T_InA_^2^*s_T_^2^_InA_/s_T_^2^__Agg_	b_T_HA_^2^ *s_T_^2^_HA_/s_T_^2^__Agg_	2*b_T_HA_*b_T_InA_*s_T_InA,HA_/s_T_^2^__Agg_
Explained by T (C + E)	0.219	0.029 (0.014–0.031)	0.133 (0.130–0.190)	0.056 (0.036–0.080)
	Total phenot.	b_G_InA_^2^*s_G_^2^_InA_/s^2^__Agg_	b_G_InA_^2^*s_G_^2^_InA_/s^2^__Agg_	2*b_G_HA_*b_G_InA_*s_G_InA,HA_/s^2^__Agg_
Explained by G (A + D)	0.297	0.009 (0.004–0.016)	0.226 (0.200–0.257)	0.061 (0.058–0.076)
		b_T_InA_^2^*s_T_^2^_InA_/s^2^__Agg_	b_T_HA_^2^ *s_T_^2^_HA_/s^2^__Agg_	2*b_T_HA_*b_T_InA_*s_T_InA,HA_/s^2^__Agg_
Explained by T (C + E)	0.062	0.008 (0.005–0.013)	0.037 (0.023–0.051)	0.016 (0.010–0.017)

Effects of sex and age closely resembled the results as obtained in the phenotypic analysis (Table 4). The regression coefficients in the A + D part of the model were 0.134 for InA (CI-95: 0.091–0.174) and 0.830 for HA (CI = 95: 0.778–0.881). In the C + E part of the model these were 0.205 for InA (CI-95: 0.149–0.268) and 0.636 for HA (CI-95: 0.532–0.744). Table 6 also contains the decomposition of standardized phenotypic Agg variance based on the regression models. In the A + D part of the model, the total explained (broad-sense) genetic variance of Agg is 41.4%, which is decomposed into 1.3% (InA; CI-95: 0.06–1.8%), 31.6% (HA; CI-95: 27.5–35.8%), and 8.5% (CI-95: 6–10.8%) due to the broad-sense genetic covariance between InA and HA. In the C + E part of the model, the total explained environmental variance was 21.9%. This is decomposed into 2.9% (InA; CI-95: 1.4–3.1%), 13.3% (HA; CI-95: 13.0–19.0%), and 5.6% (CI-95: 3.6–8.0%) due to the environmental covariance of InA and HA. At the broad sense genetic and environmental levels, HA emerged as the better predictor (genetic: 31.6%/41.4% = 76%; environmental 13.3%/21.9% = 61%).

To evaluate the predictive contributions to the phenotype variance of Agg, we standardized by the phenotypic variance (Table 6 bottom part). The total explained variance was 29.7% (A + D) + 6.2% (C + E) = 35.9%. As expected, this is almost equal to the percentage of explained variance in the phenotypic analyses (see above: 35.6%). The 35.9% is decomposed as follows. A + D contributed 0.9% due to genetic InA, 22.6% due to genetic HA, 6.1% due to the genetic covariance of InA and HA. C + E contributed 0.8% due to environmental InA, 3.7% due to environmental HA, and 1.6% due to the environmental covariance between InA and HA. By far the best predictor is genetic HA, which accounted for 22.6%/35.9% = 63% of the phenotypic variance of Agg. The remaining 37% is distributed over the other 5 remaining sources of variance.

### Combined Genetic Covariance Structure and Regression Analyses in Adults

Table 3 includes the correlations between twins and among scales in the adult twins. The MZ twin correlations were 0.46 (Agg), 0.42 (InA), and 0.36 (HA). The DZ correlations were substantially lower: 0.17 (Agg), 0.20 (InA), and 0.13 (HA). In the genetic covariance structure model, we therefore included additive genetic (A), dominance genetic (D), and unique environmental (E) components. The estimates obtained in fitting the ADE model are given in Table 5 (bottom). The narrow sense heritabilities are 21% (Agg), 31% (InA), and 12% (HA). The dominance variance components are relatively large: 26% (Agg), 13% (InA), and 19% (HA), giving rise to broad-sense heritabilities of 21 + 26 = 47% (Agg), 31 + 13 = 44% (InA), and 12 + 19 = 31% (HA). The unshared environmental variance is relatively large: 53% (Agg), 56% (InA), and 62% (HA). The estimates of the regression coefficients, for the phenotypic, genetic and non-genetic components in our model, are given in Table 7.

**Table 7:** Genetic (G(A + D)) and environmental (T(E)) regression results in adults, where for both the genetic and environmental regression ${\text{Agg}}_{{\text{i}}} = {\text{b}}_{0} + {\text{b}}_{{{\text{Sex}}}} *{\text{Sex}}_{{\text{i}}} + {\text{b}}_{{{\text{Age}}}} *{\text{Age}}_{{\text{i}}} + {\text{b}}_{{{\text{InA}}}} *{\text{InA}}_{{\text{i}}} + {\text{b}}_{{{\text{HA}}}} *{\text{HA}}_{{\text{i}}} + \varepsilon_{{\text{i}}}$

Adults	Agg	InA	HA
Raw regression coefficients with standard errors (se) or CI-95 intervals in parentheses			
Intercept (b_0_)	3.86 (se 0.126)	7.58 (se 0.133)	7.59 (se 0.123)
Sex (b_sex_)	0.722 (se 0.090)	− 0.024 (se 0.093)	0.053 (se 0.086)
Age (b_Age_)	− 0.003 (se 0.003)	− 0.036 (se 0.004)	− 0.010 (se 0.003)
G (A + D) Agg on InA (b_InA_)	0.399 (0.374–0.485)		
G (A + D) Agg on HA (b_HA_)	0.276 (0.149–0.395)		
T (E) Agg on InA (b_InA_)	0.201 (0.169–0.256)		
T (E) Agg on HA (b_HA_)	0.118 (0.055–0.176)		

Proportions of explained phenotypic variance of Agg with CI-95 intervals in parentheses				
	Total genetic	b_G_InA_^2^*s_G_^2^_InA_/s_G_^2^__Agg_	b_G_HA_^2^ *s_G_^2^_HA_/s_G_^2^__Agg_	2*b_G_HA_*b_G_InA_*s_G_InA,HA_/s_G_^2^__Agg_
Explained by G(A + D)	0.348	0.184 (0.108–0.265)	0.062 (0.050–0.110)	0.101 (0.068–0.129)
	Total environ	b_T_InA_^2^*s_T_^2^_InA_/s_T_^2^__Agg_	b_T_HA_^2^ *s_T_^2^_HA_/s_T_^2^__Agg_	2*b_T_HA_*b_T_InA_*s_T_InA,HA_/s_T_^2^__Agg_
Explained by T(E)	0.164	0.052 (0.052–0.069)	0.017 (0.016–0.040)	0.024 (0.013–0.029)
	Total phenot	b_G_InA_^2^*s_G_^2^_InA_/s^2^__Agg_	b_G_InA_^2^*s_G_^2^_InA_/s^2^__Agg_	2*b_G_HA_*b_G_InA_*s_G_InA,HA_/s^2^__Agg_
Explained by G(A + D)	0.162	0.086 (0.055–0.124)	0.029 (0.009–0.061)	0.047 (0.029–0.061)
		b_T_InA_^2^*s_T_^2^_InA_/s^2^__Agg_	b_T_HA_^2^ *s_T_^2^_HA_/s^2^__Agg_	2*b_T_HA_*b_T_InA_*s_T_InA,HA_/s^2^__Agg_
Explained by T(E)	0.049	0.027 (0.020–0.027)	0.009 (0.008–0.010)	0.013 (0.010–0.014)

The effects of sex and age closely resembled the results of the phenotypic analysis (Table 4). The regression coefficients in the A + D part of the model were 0.399 for InA (CI-95: 0.374–0.485) and 0.276 for HA (CI = 95: 0.149–0.395). In the E part of the model these were 0.201 for InA (CI-95: 0.169–0.256) and 0.118 for HA (CI-95: 0.055–0.176). Table 7 also contains the decomposition of standardized phenotype Agg variance based on the regression models. In the A + D part of the model, the total explained (broad-sense) genetic variance of Agg was 34.8%, which is decomposed into 18.4% (InA; CI-95: 10.8–26.5%), 6.2% (HA; CI-95: 5–11%), and 10.1% (CI-95: 6.8–12.9%), due to the broad-sense genetic covariance between InA and HA. In the E part of the model, the total explained environmental variance was 16.4%. This is decomposed into 5.2% (InA; CI-95: 5.2–6.9%), 1.7% (HA; CI-95: 1.6–4%), and 2.4% (CI-95: 1.3–2.9%) due to the environmental covariance of InA and HA. At the broad sense genetic and environmental levels, InA emerges as the better predictor (genetic: 18.4%/34.8% = 53%; environmental 5.2%/16.4% = 32%).

To evaluate the predictive contributions to the phenotype variance of Agg, we standardized by the phenotypic variance (Table 7 bottom part). The total explained variance was 16.2% (A + D) + 4.9% (E) = 21%. As expected this resembles the percentage of the explained variance in the phenotypic analyses (as mentioned above: 18.6%). The 21% is decomposed as follows. A + D contributes 8.6% due to genetic InA, 2.9% due to genetic HA, 4.7% due to the genetic covariance of InA and HA. E contributes 2.7% due to environmental InA, 0.9% due to environmental HA, and 1.3% due to the environmental covariance between InA and HA. By far the best predictor is genetic InA, which accounts for 8.6%/21% = 41% of the phenotypic variance of Agg.

## Discussion

In this contribution, we integrated a regression model within genetic covariance modeling. We applied the model to data from children and adults to address the question of differential prediction of aggression (Agg) by two components of ADHD, i.e. inattention (InA) and hyperactivity (HA). These types of questions of a best genetic predictor of an outcome trait or disease may come up in multiple contexts, such as the prediction of educational attainment by cognitive ability and non-cognitive skills (Demange et al. 2020) or hypertension and cardiovascular outcomes by multiple correlated factors (Lucaroni et al. 2019).

Also, the integrated model that we presented can be applied beyond the classical twin design to any genetically informative dataset or design that allows estimation of genetic and a non-genetic covariance matrices, including adoption or family studies and single-nucleotide polymorphism (SNP) based approaches to infer heritability and genetic covariance matrices from GWA studies or from their summary statistics. The possibility to consider ADCE models, rather than limiting to e.g., AE or ACE, depends on the study design and the appropriate identifying constraints. In our model for twin data these constraints involved specifying a one-factor model for the common environmental influences and the absence of genetic dominance for 1 of the 3 phenotype outcomes. We note that the present approach of estimating genetic and environmental covariance matrices, and simultaneously modeling these, differs slightly from the standard multivariate genetic covariance structure modeling where genetic and environmental covariance matrices are subjected directly to a structural equation model (e.g., a growth curve model, autoregressive model, or a common factor model). However, the present approach allows us to fit the model of interest (i.e., the regression model) to the broad-sense genetic (A + D) and the total environmental (C + E) covariance matrices, provided that these are positive definite.

Application of these methods produced a clear set of results concerning the prediction of aggression in children and in adults. In children, genetic hyperactivity was without doubt the stronger predictor of aggression, after taking into account the effects of inattention and the shared covariance of hyperactivity and inattention. A stronger predictive value of HA for aggression in children is consistent with several lines of research. There is evidence of different neural correlates of ADHD with predominantly hyperactive-impulsive, predominantly inattentive and the combined subtype (Saad et al. 2017). Hyperactivity is a stronger predictor of conduct problems than inattention in girls with ADHD (Lee and Hinshaw 2006). The information on 10-year old twins was collected from maternal ratings, and rater effects could have contributed to the results that were obtained. Vierikko et al. (2004) assessed the relation between aggression and hyperactivity/impulsivity from parents in the home situation and from teachers in the classroom. They report high genetic correlations between aggression and hyperactivity both when analyses were based on teacher and on parental ratings.

The results obtained in the analyses of adult self-ratings led to different conclusions concerning the prediction of aggression: genetic inattention clearly was the better predictor of aggression, again after considering the effects of hyperactivity and the covariance of hyperactivity and inattention. We note, however, that the adult dataset included self-report measures collected at different points in time. Still, assuming the test–retest reliability of the inattention test and the hyperactivity are about the same in adults, the difference in measurement occasion would not explain the relatively stronger role of inattention.

In conclusion, our genetic modeling of the trivariate twin data provided an insight into the genetic and non-genetic predictors of aggression. Standard Cholesky decompositions are commonly used to obtain estimates of genetic and environmental covariance matrices. We note that the Cholesky parameterization itself can be used as a regression model (with the dependent variable as the last variable; e.g., de Jong 1999). However, the present approach has the advantage of basing the regression model on the A + D and the C + E covariance matrices, which is useful if the covariances matrices (A and/or D, C and/or E) are (near) singular. In that case, we consider the option to be able to address the prediction issue at the broad-sense genetic or total environmental level to be a worthwhile one. In addition, our present approach to regression modeling in OpenMx allows a decomposition of the variance of the dependent variable (Aggression) into raw and standardized variance components. We carried out the ACDE decomposition and regression analysis separately in children and adults, with age and sex as fixed covariates. We note that the present implementation of the regression model in genetic covariance structure modeling can be extended to include fixed covariates as moderators of the regression parameters. For instance, in our adult data the mean age is ~ 30 years, but the variation in age is quite large (SD: 12.5). The present approach to the regression analysis can be extended to include age as a continuous moderator. We included the regression of aggression on HA and InA at the genetic and environmental level and obtained estimates of the phenotypic, genetic, and environmental variance components standardized by the total phenotypic variance, which revealed the relative contributions of genetic and environmental factors to the phenotypic regression of aggression on hyperactivity and inattention.

## Electronic supplementary material

Below is the link to the electronic supplementary material.

Supplementary file1 (DOCX 20 kb)
