# Supplementary material for: Genetically Informed Regression Analysis: Application to Aggression Prediction by Inattention and Hyperactivity in Children and Adults
Source: Behav Genet. 2020 Dec 1;51(3):250–63. doi: 10.1007/s10519-020-10025-9 (PMC8093158; doi:10.1007/s10519-020-10025-9)
Supplement: Supplementary file 1 — Supplementary file1 (DOCX 20 kb) [file 10519_2020_10025_MOESM1_ESM.docx]

#

# Appendix

#

#Genetically informed regression analysis:

#Application to Aggression prediction by Inattention and Hyperactivity in children and adults

#

#

#Dorret I. Boomsma, Toos CEM van Beijsterveldt, Veronika V. Odintsova, Michael C Neale, Conor V. Dolan

#

#

rm(list=ls(all=TRUE))

#

library(OpenMx) # openmx

library(MASS)

# should not make a difference.

# if you downloaded openmx from the R lib dispository, NPSOL is not available

mxOption(NULL, "Default optimizer","NPSOL")

#mxOption(NULL, "Default optimizer","CSOLNP")

#

# the acde model in the children.

#> A1

# [,1] [,2] [,3]

A=matrix(c(

16.202160, 6.699542, 7.001421,

6.699542, 2.770239, 2.895065,

7.001421, 2.895065, 3.025516),3,3)

D=matrix(c(

0, 0.000000, 0.000000,

0, 8.849712, 3.287376,

0, 3.287376, 4.404570),3,3)

C=matrix(c(

1.8652165, 0.6627402, 0.4425909,

0.6627402, 0.2354818, 0.1572594,

0.4425909, 0.1572594, 0.1050209),3,3)

E=matrix(c(

4.503955, 1.127584, 1.173916,

1.127584, 4.209324, 1.222558,

1.173916, 1.222558, 1.988946),3,3)

#

Sph=A+D+C+E

Smz12=A+D+C

Sdz12=.5*A+.25*D+C

Smz=Sdz=matrix(0,6,6)

Smz[1:3,1:3]=Smz[4:6,4:6]=Sph

Sdz[1:3,1:3]=Sdz[4:6,4:6]=Sph

Smz[1:3,4:6]=Smz[4:6,1:3]=Smz12

Sdz[1:3,4:6]=Sdz[4:6,1:3]=Sdz12

#

#

# [1] "famnr" "zyg" "age" "sex1" "sex2" "aggr1" "inatt1" "hyp1"

# [9] "aggr2" "inatt2" "hyp2"

#

Nmz=4209

Ndz=7136

datmz1=mvrnorm(Nmz,rep(0,6),Sigma=Smz, emp=T) # exact data sim

datdz1=mvrnorm(Ndz,rep(0,6),Sigma=Sdz, emp=T) # exact data sim

# add sex and age, although these have no actual effect in the simulated data

agemz=rnorm(Nmz,10,1)

agedz=rnorm(Ndz,10,1)

sexmz1=sexmz2=sample(c(0,1),Nmz,replace=T)

sexdz1=sample(c(0,1),Ndz,replace=T)

sexdz2=sample(c(0,1),Ndz,replace=T)

datmz=cbind(agemz,sexmz1,sexmz2,datmz1)

datdz=cbind(agedz,sexdz1,sexdz2,datdz1)

datmz=as.data.frame(datmz)

datdz=as.data.frame(datdz)

colnames(datmz)=colnames(datdz)= c('age','sex1','sex2','aggr1','inatt1','hyp1','aggr2','inatt2','hyp2')

#

# openmx .............................

# part 1: summary stats

# Select Variables for Analysis

#

nv <- 3 # number of variables

ntv <- nv*2 # number of total variables

selVars <- colnames(datdz)[c(4:9)] # 't1' 't2'

#

# age sex 1 sex 2 and age

covVars <- colnames(datdz)[1:3] # age sex1 and sex2

#

# SAT Model - saturated model to obtain FIML estimates of means and covariances matrices

# in MZ and DZ twins corrected for age and sex

#

RMZ <- mxMatrix( type="Stand", nrow=6, ncol=6,

free=T,

labels=c('m1','m2','m3','m4','m5','m6','m7','m8','m9','m10','m11','m12','m13','m14','m15'),

values=.4,

name="RMZ" ) # mz correlation matrix

DMZ <- mxMatrix( type="Diag", nrow=6, ncol=6,

free=c(T,T,T,T,T,T),

labels=c('ms1','ms2','ms3','ms4','ms5','ms6'),

values=c(3,3,3,3,3,3),

name="DMZ" ) # mz stdevs

#

RDZ <- mxMatrix( type="Stand", nrow=6, ncol=6,

free=T,

labels=c('d1','d2','d3','d4','d5','d6','d7','d8','d9','d10','d11','d12','d13','d14','d15'),

values=.4,

name="RDZ" ) # dz correlation matrix

DDZ <- mxMatrix( type="Diag", nrow=6, ncol=6,

free=c(T,T,T,T,T,T),

labels=c('ds1','ds2','ds3','ds4','ds5','ds6'),

values=c(3,3,3,3,3,3),

name="DDZ" ) # dz stdev

# covariance matrices

SMZ <- mxAlgebra( expression=DMZ %*%RMZ%*%t(DMZ), name="expSMZ" ) # MZ

SDZ <- mxAlgebra( expression=DDZ %*%RDZ%*%t(DDZ), name="expSDZ" ) # DZ

#

#

# Matrix & Algebra for expected means vector and expected thresholds

# Matrix & Algebra for expected means vector and expected thresholds

#

# intercept for fixed covariates sex1 sex2 and age

mean_intercept <- mxMatrix( type="Full", nrow=1, ncol=3,

free=TRUE,

labels=c("b01","b02","b03"),

values=c(0,0,0), # st vals

name="b0" )

# se and age fixed covariates a.k.a. definition variables

defAge <- mxMatrix( type="Full", nrow=1, ncol=1, free=FALSE,

labels=c("data.age"), name="Age" )

defsex1 <- mxMatrix( type="Full", nrow=1, ncol=1, free=FALSE,

labels=c("data.sex1"), name="Sex1" )

defsex2 <- mxMatrix( type="Full", nrow=1, ncol=1, free=FALSE,

labels=c("data.sex2"), name="Sex2" )

# regression coefficients age and sex

B1age <- mxMatrix( type="Full", nrow=1, ncol=3, free=TRUE,

values=.0,

label=c("ba1","ba2","ba3"), name="b1age" )

B1sex <- mxMatrix( type="Full", nrow=1, ncol=3, free=TRUE,

values=c(0,0,0),

label=c("bs1","bs2","bs3"), name="b1sex" )

# the means

correctedMean <- mxAlgebra( expression=

cbind(b0+(b1age%x%Age+b1sex%x%Sex1),

b0+(b1age%x%Age+b1sex%x%Sex2)),

name="correctedMean" )

#

# the data

#

dataMZ <- mxData( observed=datmz, type="raw" )

dataDZ <- mxData( observed=datdz, type="raw" )

#

#

# Expectation objects for Multiple Groups

expMZ <- mxExpectationNormal( covariance="expSMZ", means="correctedMean", dimnames=selVars)

expDZ <- mxExpectationNormal( covariance="expSDZ", means="correctedMean", dimnames=selVars)

pars <- list( RMZ, DMZ, RDZ, DDZ )

bits <- c(mean_intercept, defAge, defsex1, defsex2, B1sex, B1age, correctedMean)

#

funML <- mxFitFunctionML()

modelMZ <- mxModel( pars, bits, SMZ, dataMZ, funML,expMZ, name="MZ" )

modelDZ <- mxModel( pars, bits, SDZ, dataDZ, funML,expDZ, name="DZ" )

#

# Create Confidence Interval Objects

ciCov <- mxCI( c('m3','m8','m12','d3','d8','d12'))

#

# Combine Groups

multi <- mxFitFunctionMultigroup( c("MZ","DZ") )

SATModel <- mxModel( "SAT", pars, modelMZ, modelDZ, funML, multi, ciCov )

#

# ------------------------------------------------------------------------------

# RUN MODEL

#

SATFit <- mxRun(SATModel, intervals=F) # to get the confidence intervals: interval=T

#SATFit = mxTryHard(SATModel, 100) # try multple starting values if you like

SATsum <- summary(SATFit)

# see results correlation matrices and stdev mz

round(SATFit$RMZ$values,3)

round(diag(SATFit$DMZ$values),3)

# see results correlation matrices and stdev dz

round(SATFit$RDZ$values,3)

round(diag(SATFit$DDZ$values),3)

#

# openmx part 2

#

# ACDE model....

# ------------------------------------------------------------------------------

# first fit the model to the continuous data

# PREPARE MODEL continuous data

#

# ACE Model

# Matrices declared to store a, c, and e Path Coefficients

#

pathA <- mxMatrix( type="Lower", nrow=3, ncol=3,

free=c(T,T,T,T,T,T), # full A cholesky

values=c(2,2,2,0,0,0),

label=c("a11","a21","a31","a22","a32","a33"),

name="a" )

pathC <- mxMatrix( type="Lower", nrow=3, ncol=3,

free=c(T,T,T,F,F,F), # C cholesky limioted to 3 parameters

values=c(.7,.7,.7,0,0,0),

label=c("c11","c21","c31","c22","c32","c33"),

name="c" )

pathD <- mxMatrix( type="Lower", nrow=3, ncol=3,

free=c(F,F,F,T,T,T), # D cholesky limited to last two variables

values=c(0,0,0,2,1,2),

label=c("d11","d21","d31","d22","d32","d33"),

name="d" )

pathE <- mxMatrix( type="Lower", nrow=3, ncol=3,

free=c(T,T,T,T,T,T), # full E cholesky

values=c(2,0,0,2,0,2),

label=c("e11","e21","e31","e22","e32","e33"),

name="e" )

#

# Matrices generated to hold A, C, and E computed Variance Components

# Cholesky decomp -> A, C, D, E cov matrices

covA <- mxAlgebra( expression=a %*% t(a), name="A" )

covC <- mxAlgebra( expression=c %*% t(c), name="C" )

covE <- mxAlgebra( expression=e %*% t(e), name="E" )

covD <- mxAlgebra( expression=d %*% t(d), name="D" )

#

#

mean_intercept <- mxMatrix( type="Full", nrow=1, ncol=3,

free=TRUE,

labels=c("b01","b02","b03"),

values=c(0,0,0),

name="b0" )

# fixed covariates (a.k.a. definition variables)

defAge <- mxMatrix( type="Full", nrow=1, ncol=1, free=FALSE,

labels=c("data.age"), name="Age" )

defsex1 <- mxMatrix( type="Full", nrow=1, ncol=1, free=FALSE,

labels=c("data.sex1"), name="Sex1" )

defsex2 <- mxMatrix( type="Full", nrow=1, ncol=1, free=FALSE,

labels=c("data.sex2"), name="Sex2" )

# regression coefficients

B1age <- mxMatrix( type="Full", nrow=1, ncol=3, free=TRUE,

values=.0,

label=c("ba1","ba2","ba3"), name="b1age" )

B1sex <- mxMatrix( type="Full", nrow=1, ncol=3, free=TRUE,

values=c(0,0,0),

label=c("bs1","bs2","bs3"), name="b1sex" )

# conditional means (sex age )

correctedMean <- mxAlgebra( expression=

cbind(b0+(b1age%x%Age+b1sex%x%Sex1),

b0+(b1age%x%Age+b1sex%x%Sex2)),

name="correctedMean" )

# phenotypic cov matrix

covP <- mxAlgebra( expression=A+C+D+E, name="V" )

# Algebras generated to hold Parameter Estimates and Derived Variance Components

colVars <- rep(c('A','C','D','E','SA','SC','SD','SE'),each=nv)

estVars <- mxAlgebra( expression=cbind(A,C,D,E,A/V,C/V,D/V,E/V), name="Vars")

#

# Algebra for expected Variance/Covariance Matrices in MZ & DZ twins

#

covMZ <- mxAlgebra( expression= rbind( cbind(V , A+C+D),

cbind(A+C+D , V)), name="expCovMZ" )

covDZ <- mxAlgebra( expression= rbind( cbind(V, 0.5%x%A+.25%x%D+C),

cbind(0.5%x%A+.25%x%D+C , V)), name="expCovDZ" )

#

# data

#

dataMZ <- mxData( observed=datmz, type="raw" )

dataDZ <- mxData( observed=datdz, type="raw" )

#

#

# Expectation objects for Multiple Groups

expMZ <- mxExpectationNormal( covariance="expCovMZ", means="correctedMean", dimnames=selVars)

expDZ <- mxExpectationNormal( covariance="expCovDZ", means="correctedMean", dimnames=selVars)

pars <- list( pathA, pathC, pathD, pathE, covA, covC, covD, covE, covP, estVars )

bits <- c(mean_intercept, defAge, defsex1, defsex2, B1sex, B1age, correctedMean)

#

funML <- mxFitFunctionML()

modelMZ <- mxModel( pars, bits, covMZ, dataMZ, funML,expMZ, name="MZ" )

modelDZ <- mxModel( pars, bits, covDZ, dataDZ, funML,expDZ, name="DZ" )

#

#

# Combine Groups

multi <- mxFitFunctionMultigroup( c("MZ","DZ") )

AcdeModel <- mxModel( "ACDE", pars, modelMZ, modelDZ, funML, multi )

#

# ------------------------------------------------------------------------------

# ------------------------------------------------------------------------------

#AcdeFitCont <- mxTryHard(AcdeModel, 50)

#

AcdeFitCont <- mxRun(AcdeModel)

AcdeSummCont <- summary(AcdeFitCont)

AcdeSummCont

mxCompare(SATFit, AcdeFitCont)

#

# extract results

A1=AcdeFitCont$A$result

D1=AcdeFitCont$D$result

C1=AcdeFitCont$C$result

E1=AcdeFitCont$E$result

A1+D1+C1+E1 -> Ph1

A1

D1

C1

E1

cov2cor(A1)

cov2cor(D1)

cov2cor(C1)

cov2cor(E1)

A1/Ph1

D1/Ph1

C1/Ph1

E1/Ph1

#

#

# do the phenotypic regression analysis in openmx this is based on the phenotyipc 3x3 matrix

#

# ------------------------------------------------------------------------------

# covariance matrix

pathPh <- mxMatrix( type="Lower", nrow=3, ncol=3,

free=c(T,T,T,T,T,T),

values=c(4,0,0,4,0,4),

label=c("f11","f21","f31","f22","f32","f33"),

name="ph" )

# mz off diag block

OffdiagM <- mxMatrix( type="Full", nrow=3, ncol=3,

free=c(T,T,T,T,T,T,T,T,T),

values=c(4,4,4,4,4,4,4,4,4),

name="OffM" )

# dz offdiag block

OffdiagD <- mxMatrix( type="Full", nrow=3, ncol=3,

free=c(T,T,T,T,T,T,T,T,T),

values=c(4,4,4,4,4,4,4,4,4),

name="OffD" )

##

covPh <- mxAlgebra( expression=ph %*% t(ph), name="SPH" ) # 3x3 phenotypic cov matrix

# calculate the regression coefficients

bph <- mxAlgebra(expression=solve(SPH[2:3,2:3])%*%SPH[2:3,1], name='bPH')

# get the decomposition of variance in aggression

part1=mxAlgebra(expression=bPH[1,1]*bPH[1,1]*SPH[2,2],name="PHp1")

part2=mxAlgebra(expression=bPH[2,1]*bPH[2,1]*SPH[3,3],name="PHp2")

part12=mxAlgebra(expression=2*bPH[1,1]*bPH[2,1]*SPH[3,2],name="PHp12") # due to cov inatt, hyp

# decomposition of variance in components %

Phexpl1=mxAlgebra(cbind(PHp1,PHp2,PHp12)/SPH[1,1], name='PHexpl1')

#

# sex and age as fixed covariates (a.k.a. definition variables)

mean_intercept <- mxMatrix( type="Full", nrow=1, ncol=3,

free=TRUE,

labels=c("b01","b02","b03"),

values=c(0,0,0),

name="b0")

# revise ............................................ revise add sex as cov

defAge <- mxMatrix( type="Full", nrow=1, ncol=1, free=FALSE,

labels=c("data.age"), name="Age" )

defsex1 <- mxMatrix( type="Full", nrow=1, ncol=1, free=FALSE,

labels=c("data.sex1"), name="Sex1" )

defsex2 <- mxMatrix( type="Full", nrow=1, ncol=1, free=FALSE,

labels=c("data.sex2"), name="Sex2" )

B1age <- mxMatrix( type="Full", nrow=1, ncol=3, free=TRUE,

values=.0,

label=c("ba1","ba2","ba3"), name="b1age" )

B1sex <- mxMatrix( type="Full", nrow=1, ncol=3, free=TRUE,

values=c(0,0,0),

label=c("bs1","bs2","bs3"), name="b1sex" )

#

correctedMean <- mxAlgebra( expression=

cbind(b0+(b1age%x%Age+b1sex%x%Sex1),

b0+(b1age%x%Age+b1sex%x%Sex2)),

name="correctedMean" )

#

# cov matrix 6x6 in MZ and DZ

#

covMZ <- mxAlgebra( expression= rbind( cbind(SPH , OffM),

cbind(t(OffM),SPH)), name="expCovMZ" )

covDZ <- mxAlgebra( expression= rbind( cbind(SPH, OffD),

cbind(t(OffD), SPH)), name="expCovDZ" )

#

dataMZ <- mxData( observed=datmz, type="raw" )

dataDZ <- mxData( observed=datdz, type="raw" )

#

# Expectation objects for Multiple Groups

expMZ <- mxExpectationNormal( covariance="expCovMZ", means="correctedMean", dimnames=selVars)

expDZ <- mxExpectationNormal( covariance="expCovDZ", means="correctedMean", dimnames=selVars)

#

pars <- list(pathPh, covPh, bph, part1,part2,part12,Phexpl1)

bits <- c(mean_intercept, defAge, defsex1, defsex2, B1sex, B1age, correctedMean)

#

funML <- mxFitFunctionML()

modelMZ <- mxModel( bits, pars, OffdiagM, covMZ, dataMZ, funML,expMZ, name="MZ" )

modelDZ <- mxModel( bits, pars, OffdiagD, covDZ, dataDZ, funML,expDZ, name="DZ" )

#

# Create Confidence Interval Objects

ciPAR1 <- mxCI("PHexpl1")

ciPAR2 <-mxCI("bPH")

#'PHexpl1'

# Phexpl1=mxAlgebra(cbind(PHp1,PHp2,PHp12)/SPH[1,1], name='PHexpl1')

#

# Combine Groups

multi <- mxFitFunctionMultigroup( c("MZ","DZ") )

RegModelPH <- mxModel( "regPheno", pars,modelMZ, modelDZ, funML, multi,

ciPAR1,ciPAR2)

#

# ------------------------------------------------------------------------------

# ------------------------------------------------------------------------------

# RUN MODELS

# Run ACE Model

#

RegFitContPH <- mxRun(RegModelPH, intervals=F)

RegSummContPH <- summary(RegFitContPH)

RegSummContPH

round(RegFitContPH@output$estimate,4)

#

# ------------------------------------------------------------------------------------

#

# ACDE regression regression based on A+D and based on C+E

# ------------------------------------------------------------------------------

#

# ACE Model

# Matrices declared to store a, c, and e Path Coefficients

#

pathA <- mxMatrix( type="Lower", nrow=3, ncol=3,

free=c(T,T,T,T,T,T),

values=c(2,2,2,0,0,0),

label=c("a11","a21","a31","a22","a32","a33"),

name="a" )

pathC <- mxMatrix( type="Lower", nrow=3, ncol=3,

free=c(T,T,T,F,F,F),

values=c(.7,.7,.7,0,0,0),

label=c("c11","c21","c31","c22","c32","c33"),

name="c" )

pathD <- mxMatrix( type="Lower", nrow=3, ncol=3,

free=c(F,F,F,T,T,T),

values=c(0,0,0,2,1,2),

label=c("d11","d21","d31","d22","d32","d33"),

name="d" )

pathE <- mxMatrix( type="Lower", nrow=3, ncol=3,

free=c(T,T,T,T,T,T),

values=c(2,0,0,2,0,2),

label=c("e11","e21","e31","e22","e32","e33"),

name="e" )

#

# Matrices generated to hold A, C, and E computed Variance Components

#

covA <- mxAlgebra( expression=a %*% t(a), name="A" )

covC <- mxAlgebra( expression=c %*% t(c), name="C" )

covE <- mxAlgebra( expression=e %*% t(e), name="E" )

covD <- mxAlgebra( expression=d %*% t(d), name="D" )

# A+D and C+D matrices

covAD <-mxAlgebra(expression=A+D, name="SAD")

covCE <-mxAlgebra(expression=C+E, name="SCE")

# regression coefficients

bAD <- mxAlgebra(expression=solve(SAD[2:3,2:3])%*%SAD[2:3,1], name='bAD')

bCE <- mxAlgebra(expression=solve(SCE[2:3,2:3])%*%SCE[2:3,1], name='bCE')

# decomposition of variance (A+D)

partAD1=mxAlgebra(expression=bAD[1,1]*bAD[1,1]*SAD[2,2],name="AD1p1")

partAD2=mxAlgebra(expression=bAD[2,1]*bAD[2,1]*SAD[3,3],name="AD1p2")

partAD12=mxAlgebra(expression=2*bAD[1,1]*bAD[2,1]*SAD[3,2],name="AD1p12")

# decomposition of variance (E+C)

partCE1=mxAlgebra(expression=bCE[1,1]*bCE[1,1]*SCE[2,2],name="CE1p1")

partCE2=mxAlgebra(expression=bCE[2,1]*bCE[2,1]*SCE[3,3],name="CE1p2")

partCE12=mxAlgebra(expression=2*bCE[1,1]*bCE[2,1]*SCE[3,2],name="CE1p12")

#

# Standardized variance components

ADexpl1=mxAlgebra(cbind(AD1p1,AD1p2,AD1p12)/SAD[1,1], name='ADexpl1') # standardized by A+D

ADexpl2=mxAlgebra(cbind(AD1p1,AD1p2,AD1p12)/(SAD[1,1]+SCE[1,1]), name='ADexpl2') # standardized by phenotypic totoal

CEexpl1=mxAlgebra(cbind(CE1p1,CE1p2,CE1p12)/SCE[1,1], name='CEexpl1') # standardized by E+C

CEexpl2=mxAlgebra(cbind(CE1p1,CE1p2,CE1p12)/(SAD[1,1]+SCE[1,1]), name='CEexpl2') # standardized by phenotypic total

#

varExplAD1=mxAlgebra(expression=(t(bAD)%*%SAD[2:3,2:3]%*%(bAD))/SAD[1,1], name='ADexpv1') # total explained A+D

varExplCE1=mxAlgebra(expression=(t(bCE)%*%SCE[2:3,2:3]%*%(bCE))/SCE[1,1], name='CEexpv1') # total explained C+E

#

varExplAD2=mxAlgebra(expression=(t(bAD)%*%SAD[2:3,2:3]%*%(bAD))/(SAD[1,1]+SCE[1,1]), name='ADexpv2') # total explain (% of tot variance)

varExplCE2=mxAlgebra(expression=(t(bCE)%*%SCE[2:3,2:3]%*%(bCE))/(SAD[1,1]+SCE[1,1]), name='CEexpv2')# total explain (% of tot variance)

#

#

# Matrix & Algebra for expected means vector and expected thresholds

# Matrix & Algebra for expected means vector and expected thresholds

#

#

mean_intercept <- mxMatrix( type="Full", nrow=1, ncol=3,

free=TRUE,

labels=c("b01","b02","b03"),

values=c(0,0,0),

name="b0" )

# fixed covariates (a.k.a. definition variables

defAge <- mxMatrix( type="Full", nrow=1, ncol=1, free=FALSE,

labels=c("data.age"), name="Age" )

defsex1 <- mxMatrix( type="Full", nrow=1, ncol=1, free=FALSE,

labels=c("data.sex1"), name="Sex1" )

defsex2 <- mxMatrix( type="Full", nrow=1, ncol=1, free=FALSE,

labels=c("data.sex2"), name="Sex2" )

B1age <- mxMatrix( type="Full", nrow=1, ncol=3, free=TRUE,

values=.0,

label=c("ba1","ba2","ba3"), name="b1age" )

B1sex <- mxMatrix( type="Full", nrow=1, ncol=3, free=TRUE,

values=c(0,0,0),

label=c("bs1","bs2","bs3"), name="b1sex" )

#

correctedMean <- mxAlgebra( expression=

cbind(b0+(b1age%x%Age+b1sex%x%Sex1),

b0+(b1age%x%Age+b1sex%x%Sex2)),

name="correctedMean" )

#

# Create Algebra for expected Threshold Matrices

covP <- mxAlgebra( expression=A+C+D+E, name="V" )

# Algebras generated to hold Parameter Estimates and Derived Variance Components

colVars <- rep(c('A','C','D','E','SA','SC','SD','SE'),each=nv)

estVars <- mxAlgebra( expression=cbind(A,C,D,E,A/V,C/V,D/V,E/V), name="Vars")

#

# Algebra for Variance/Covariance Matrices in MZ & DZ twins

#

covMZ <- mxAlgebra( expression= rbind( cbind(V , A+C+D),

cbind(A+C+D , V)), name="expCovMZ" )

covDZ <- mxAlgebra( expression= rbind( cbind(V, 0.5%x%A+.25%x%D+C),

cbind(0.5%x%A+.25%x%D+C , V)), name="expCovDZ" )

#

#

# data

dataMZ <- mxData( observed=datmz, type="raw" )

dataDZ <- mxData( observed=datdz, type="raw" )

#

# Expectation objects for Multiple Groups

expMZ <- mxExpectationNormal( covariance="expCovMZ", means="correctedMean", dimnames=selVars)

expDZ <- mxExpectationNormal( covariance="expCovDZ", means="correctedMean", dimnames=selVars)

pars <- list(pathA, pathC, pathD, pathE,

covA, covC, covD, covE, covP,

covAD, covCE, bAD, bCE, estVars,

partAD1,partAD2,partAD12,partCE1,partCE2,partCE12,

varExplAD1,varExplCE1,varExplAD2,varExplCE2,

ADexpl1, ADexpl2, CEexpl1, CEexpl2)

bits <- c(mean_intercept, defAge, defsex1, defsex2, B1sex, B1age, correctedMean)

#

funML <- mxFitFunctionML()

modelMZ <- mxModel( pars, bits, covMZ, dataMZ, funML,expMZ, name="MZ" )

modelDZ <- mxModel( pars, bits, covDZ, dataDZ, funML,expDZ, name="DZ" )

#

# Create Confidence Interval Objects

ciPAR1 <- mxCI("ADexpl1")

ciPAR2 <- mxCI("ADexpl2")

ciPAR3 <- mxCI("CEexpl1")

ciPAR4 <- mxCI("CEexpl2")

ciPAR5 <-mxCI(c("bAD","bCE"))

#ciPAR6 <-mxCI("bCE")

#

# Combine Groups

multi <- mxFitFunctionMultigroup( c("MZ","DZ") )

RegModel <- mxModel( "regACDE", pars, modelMZ, modelDZ, funML, multi,

ciPAR1, ciPAR2, ciPAR3, ciPAR4, ciPAR5, ciPAR6)

#

# ------------------------------------------------------------------------------

# ------------------------------------------------------------------------------

# RUN MODELS

# Run ACE Model

#

RegFitCont <- mxRun(RegModel, intervals=FALSE)

RegSummCont <- summary(RegFitCont)

RegSummCont

round(RegFitCont@output$estimate,4)

#

#RegModel <- mxModel( "regACDE", pars, modelMZ, modelDZ, funML, multi,

# ciPAR5)

#RegFitCont <- mxRun(RegModel, intervals=TRUE)
